# Chest configuration in children and adolescents with infantile nephropathic cystinosis compared with other chronic kidney disease entities and its clinical determinants

**DOI:** 10.1007/s00467-023-06058-x

**Published:** 2023-07-07

**Authors:** Sophia Müller, Rika Kluck, Celina Jagodzinski, Malina Brügelmann, Katharina Hohenfellner, Anja Büscher, Markus J. Kemper, Kerstin Fröde, Jun Oh, Heiko Billing, Julia Thumfart, Lutz T. Weber, Birgit Acham-Roschitz, Klaus Arbeiter, Burkhard Tönshoff, Martina Hagenberg, Leo Pavičić, Dieter Haffner, Miroslav Zivicnjak

**Affiliations:** 1 Department of Pediatric Kidney, Liver and Metabolic Diseases, Hannover Medical School, Children’s Hospital, Carl-Neuberg-Str. 1, 30625 Hannover, Germany; 2 Division of Pediatric Nephrology, Children’s Hospital, Rosenheim, Germany; 3 Department of Pediatrics II, University Hospital Essen, Essen, Germany; 4 Asklepios Hospital, Hamburg, Germany; 5 Division of Pediatric Nephrology, University Children’s Hospital Hamburg, Hamburg, Germany; 6 Clinic for Pediatric and Adolescent Medicine, RHK Clinic Ludwigsburg, Ludwigsburg, Germany; 7 Department of Pediatric Gastroenterology, Nephrology and Metabolic Diseases, Charité-Universitätsmedizin Berlin, Berlin, Germany; 8 Pediatric Nephrology, Children’s and Adolescents’ Hospital, University of Cologne, Faculty of Medicine and University Hospital, Cologne, Germany; 9 Department of Pediatrics, Medical University Graz, Graz, Austria; 10 Division of Pediatric Nephrology and Gastroenterology, Medical University Vienna, Vienna, Austria; 11 Department of Pediatrics I, University Children’s Hospital Heidelberg, Heidelberg, Germany; 12 Children’s Hospital St. Elisabeth and St. Barbara, Halle (Saale), Germany; 13 Zagreb, Croatia

**Keywords:** Infantile nephropathic cystinosis, Chest, Biacromial diameter, Anterior–posterior chest diameter, Chronic kidney disease, Fanconi syndrome

## Abstract

**Background:**

Infantile nephropathic cystinosis (INC) is a systemic lysosomal storage disease causing intracellular cystine accumulation, resulting in renal Fanconi syndrome, progressive kidney disease (CKD), rickets, malnutrition, and myopathy. An INC-specific disproportionately diminished trunk length compared to leg length poses questions regarding the functionality of the trunk.

**Methods:**

Thus, we prospectively investigated thoracic dimensions and proportions, as well as their clinical determinants in 44 pediatric patients with INC with CKD stages 1–5 and 97 age-matched patients with CKD of other etiology between the ages of 2–17 years. A total of 92 and 221 annual measurements of patients with INC and CKD, respectively, were performed, and associations between anthropometric and clinical parameters were assessed using linear mixed-effects models.

**Results:**

Patients with INC exhibited altered chest dimensions that were distinct from CKD controls, characterized by markedly increased chest depth to height and chest depth to chest width ratio *z*-scores (> 1.0), while those of patients with CKD were only mildly affected (*z*-score within ± 1.0). Ratio *z*-scores differed significantly between both patient groups from 2–6 years of age onward. The degree of chest disproportion in INC patients was significantly associated with both the degree of CKD and tubular dysfunction (e.g., low serum phosphate and bicarbonate) across three different age groups (2–6, 7–12, and 13–17 years).

**Conclusion:**

Our data show an INC-specific alteration in thoracic shape from early childhood onward, which is distinct from CKD of other etiologies, suggesting early childhood subclinical changes of the musculoskeletal unit of the thoracic cage, which are associated with kidney function.

**Graphical abstract:**

**Figure:**
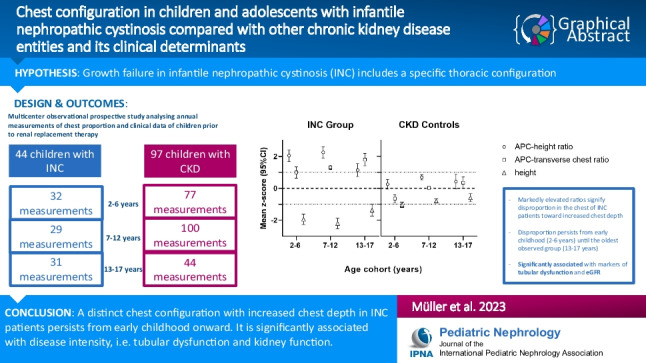
A higher resolution version of the Graphical abstract is available as Supplementary information

**Supplementary Information:**

The online version contains supplementary material available at 10.1007/s00467-023-06058-x.

## Introduction

Infantile nephropathic cystinosis (INC) is a lysosomal storage disease causing multisystem intracellular cystine crystal accumulation and ensuing multisystem complications, often first affecting the kidney [1–3]. Symptoms due to generalized proximal tubule dysfunction (Fanconi syndrome), i.e., polyuria, failure to thrive and hypophosphatemic rickets, typically manifest within the first 18 months of life, followed by progressive chronic kidney disease (CKD) and a multitude of other complications including malnutrition, myopathy, and endocrine dysfunction [1, 2, 4–6]. Myopathy and associated respiratory dysfunction are frequent features in older patients with INC, with reported aberrations in chest shape in affected patients [7–9].

Despite adequate treatment of Fanconi syndrome and cystine depleting therapy, which was shown to delay the need for kidney replacement therapy, children with INC are prone to progressive disproportionate short stature [10, 11]. This is characterized by a shift from a trunk length preserving pattern shared with children with CKD of other causes to an INC-specific leg-focused growth pattern [10], prompting further examination of the morphology of the trunk of INC patients.

Biacromial diameter, for one, is known to be linked to quality of living conditions or level of physical activity [12, 13], and the ribcage, on the other hand, can be influenced through rickets [14], which is a hallmark in INC patients [1, 4, 5]. Thus, we hypothesized that children with INC present with characteristic changes of the aforementioned dimensions when compared to their peers with CKD. To test this, we prospectively investigated thoracic dimensions and proportions, i.e., chest depth/height and chest depth/chest width ratios, in conjunction with detailed biochemical parameters. Those were assessed in a cohort of pediatric patients with INC with CKD stages 1–5 and matched CKD controls with other hereditary or congenital kidney diseases across three age groups (ages 2–6, 7–12, and 13–17 years).

## Material and methods

### Study design and patients

This analysis includes children with INC and hereditary or congenital CKD aged 2 to 17 years with CKD stages 1–5 only prior to kidney replacement therapy who are enrolled in the prospective multicenter observational cohort study “Growth and cognitive-motor abilities in children with nephropathic cystinosis and chronic kidney disease” [10, 15]. Patients with complex or syndromic diseases were excluded. Between January 2016 and January 2022, a total of 44 patients with INC and 97 age-matched CKD controls from thirteen pediatric centers across Germany and Austria were eligible for analysis. Underlying kidney diseases in the CKD control group included congenital anomalies of the kidney and urinary tract (CAKUT, 64.9%), nephronophthisis (3.1%), autosomal recessive polycystic kidney disease (ARPKD, 6.2%), and other causes of CKD (23.7%). The mean patient age was 9.94 years in patients with INC (95% CI 9.09–10.78) and 9.13 in CKD controls (95% CI 8.57–9.70; Table 1). Patients were annually assessed including physical examination, history of medication, routine biochemical parameters, and detailed anthropometric assessment. All patients regularly received dietary advice by a dietician to ensure adequate caloric and protein intake.

**Table 1:** Patient characteristics and treatment in 44 children with infantile nephropathic cystinosis with CKD stages 1–5 and 97 CKD controls

	INC		CKD		*p*-value
	Incidence %	*N*	Incidence %	*N*	
Male sex	54.5	24 of 44*	67.0	65 of 97*	0.188
Congenital CKD	–	–	89.7	87 of 97*	n.a
SGA history	18.2	6 of 33*	20.7	17 of 82*	1.000
Metabolic acidosis	40.5	30 of 74	36.2	77 of 213	0.577
Hypokalemia	44.8	39 of 87	4.4	9 of 206	0.000
Hypophosphatemia	21.7	18 of 83	2.4	5 of 208	0.000
Hypocalcemia	60.8	48 of 79	35.6	74 of 208	0.000
Anemia	15.7	14 of 89	26.0	57 of 219	0.054
Medication					
Erythropoietin	20.2	18 of 89	25.9	55 of 212	0.307
Iron	21.3	19 of 89	30.2	64 of 212	0.123
Active vitamin D	50.6	45 of 89	33.5	71 of 212	0.006
Native vitamin D	74.2	66 of 89	72.6	154 of 212	0.887
Bicarbonate	70.8	63 of 89	47.2	100 of 212	0.000
Potassium	79.8	71 of 89	1.9	4 of 212	0.000
Calcium	12.4	11 of 89	1.9	4 of 212	0.000
Phosphate	66.3	59 of 89	1.9	4 of 210	0.000
Carnitine	53.4	47 of 88	0.0	0 of 213	0.000
Antihypertensives	27.0	24 of 89	57.1	124 of 217	0.000
Growth hormone	52.3	23 of 44*	25.8	25 of 97*	0.004
Cysteamine	100.0	91 of 91	–		n.a

*N* describes the number of either patients (for static patient dependent characteristics, signified with *) or yearly measurements (for repeated clinical measurements and medication) out of overall valid patient cases or valid yearly measurements, i.e. without separation into age subgroups

*CKD* chronic kidney disease, *n.a.* not applicable, *SGA* small for gestational age

Appropriate Ethics Committee approval was obtained from the institutional review boards at each study site, and this study was performed in accordance with the Declaration of Helsinki. Written informed consent was obtained from all parents/guardians, with consent or assent from patients when appropriate for their age.

### Methods

Yearly anthropometric assessments were performed according to the International Biological Program recommendations and performed by the same investigator (M.Ž.) with standardized equipment as previously described [16–18]. An average of 2.22 yearly measurements were performed per patient, including height and thoracic parameters, i.e., biacromial diameter (shoulder width), anterior–posterior (AP) chest diameter (chest depth), and transverse chest diameter (chest width). This was used to calculate chest depth/height ratio (APC-height ratio, i.e. $\frac{APC\;in\;mm}{height\;in\;mm} \times 100$) and chest depth/chest width ratio (APC-transverse chest ratio, i.e. $\frac{APC\;in\;mm}{transverse\;chest\;diameter\;in\;mm}\;\times 100$), as measures of chest proportion. From those parameters and ratios, age- and sex-dependent z-scores were calculated using anthropometric parameters from reference data derived from healthy children [17, 18], (e.g.$\frac{\left(patient\;APC\;height\;ratio\;-\;mean\;APC\;height\;ratio\;of\;reference\;group\right)}{standard\;deviation\;of\;APC\;height\;ratio\;of\;reference\;group}=APC-height\;ratio\;z\;score$).

Information regarding current biochemical parameters and medication was obtained at each anthropometric measurement appointment. Standard laboratory techniques were used for the measurement of serum concentrations of creatinine, urea, calcium, phosphate, potassium, albumin, bicarbonate, intact parathyroid hormone (PTH), and hemoglobin blood levels. Serum calcium levels were corrected regarding albumin [19]. Estimated glomerular filtration rate (eGFR) was calculated by use of the revised Schwartz equation [20]. Intracellular leukocyte cystine levels were measured at laboratories of Hanover Medical School and the University Children’s Hospital Muenster [21, 22] and uniformly converted to nanomoles half-cystine per milligram protein. Age- and sex-dependent reference intervals were used for determining frequencies of hypokalemia, hypophosphatemia, hypocalcemia, and anemia. Metabolic acidosis was defined by presence of serum bicarbonate < 22 mmol/L [23–26].

Information from patients’ standardized pregnancy and birth health care booklets was used to classify patients born small for gestational age (SGA), i.e., if birth weight and/or birth length were below the 10^th^ percentile when compared to the respective gestational age and sex of national birth and growth data [27].

### Statistical analysis

Data presentation in yearly age cohorts (e.g., age 2 = 2.00–2.99 years) was not feasible due to the low number of patients. Therefore, patients’ measurements were divided into three age groups: (i) ages 2–6 years (109 total measurements, 77 CKD, 32 INC), (ii) ages 7–12 years (129 measurements, 100 CKD, 29 INC), and (iii) ages 13–17 years (75 measurements, 44 CKD, 31 INC). These age ranges reflect relevant stages of biological development, i.e., early childhood, mid-late childhood, and adolescence, and account for the usually slightly delayed onset of puberty in CKD patients [28]. The separation of repeated patient measurements into the age groups results in some patients being measured multiple times in the same age group and across different age groups. To adjust for the amount each patient was measured in each age group, adjustments were made to the linear mixed-effects models (see below).

Differences in categorical variables (i.e., incidence) between groups (CKD vs. INC) were analyzed using the chi-square test or Fisher’s test of significance, as appropriate. Data distribution normality of continuous data with non-repeated measurements was evaluated using the Kolmogorov–Smirnov test with and without Lilliefors correction and the Shapiro–Wilk test. For comparison of continuous variables between two groups, either the *t*-test or Mann–Whitney test were used, as appropriate. Linear mixed-effects models (MIXED procedure in SPSS) were used.

Descriptive data are displayed as appropriate, as either mean with 95% confidence interval (CI), median with interquartile range (IQR), number of data points that display respective characteristic (*n*) with percentage of overall measurements, or estimated marginal mean with 95% CI for repeated measurements.

To assess differences between patients with CKD and INC across all age groups for parameters with repeated measurements, linear mixed-effects models were used. The same method was also used to analyze the difference in anthropometric and clinical parameters between patient groups separately for the three age groups. Differences between different age groups within the same patient group (CKD or INC) were analyzed using pairwise comparison with linear mixed-effects models.

Linear mixed-effects models were further used to analyze the association of clinical parameters (covariates) with APC-height ratio and APC-chest transverse ratio separately for both patient groups. The following clinical parameters were defined as covariates: serum phosphate, calcium, potassium, HCO_3_, intact parathyroid hormone (PTH), hemoglobin blood levels, and estimated glomerular filtration rate (eGFR). For graphical representation, linear mixed-effects models were used to calculate predicted values from the repeated measurements, adjusted for multiple comparisons. For the youngest INC age group (2–6 years), linear mixed-effects models were used to perform an analysis of APC-height ratio *z*-scores and their association with eGFR nested with patient age. For all linear mixed-effects model analyses, different covariate structure models were tested and the most appropriate model was chosen according to information criteria for each analysis and group of parameters. Results were considered significant at a level of *p* < 0.05. SPSS for Windows, version 27.0 (IBM Corporation, NY, USA), was used. Graphs were generated using GraphPad Prism 9.0.0 (GraphPad Software, Inc., San Diego, CA).

## Results

### Patient characteristics

The characteristics of 44 patients with INC and 97 controls with CKD are given in Tables 1 and 2. The mean age and sex distribution did not differ between patients with INC and CKD, either across all age groups or for individual age subgroups (each *p* > 0.05). Patients with INC were diagnosed at a median age of 1.04 years (IQR 0.7, 2.1), and all received cysteamine treatment with a median dosage of 1.3 g/m^2^ body surface area (IQR 1.02, 1.68) at the time of their most recent measurement, which was started at a median age of 1.22 years (IQR 0.93, 2.48). Median leukocyte cystine levels of all patients with INC were 0.29 nmol half-cystine per milligram protein (IQR 0.15, 0.52). Antihypertensives were used more frequently in CKD controls (each *p* < 0.01, Table 1), as were erythropoietin (25.9% CKD vs. 20.2% INC) and iron substitution (30.2% CKD vs. 21.3% INC), though not significantly. Patients with INC received treatment with recombinant human growth hormone (rhGH) more frequently, as well as medication to correct the consequences of Fanconi syndrome, including supplementation of potassium, calcium, and phosphate, as well as bicarbonate and active vitamin D (each *p* < 0.01). Despite this, patients with INC showed significantly lower mean serum levels of potassium, calcium, and phosphate, as well as higher rates of hypokalemia, hypocalcemia, and hypophosphatemia compared to CKD controls (each *p* < 0.01, Tables 1 and 2). When evaluating all observed patients (2–17 years), mean eGFRcr was significantly lower in CKD controls compared to patients with INC (48 mL/min/1.73 m^2^ (95% CI 44–52) versus 66 mL/min/1.73 m^2^ (95% CI 60–72), *p* < 0.001), as were hemoglobin levels (12.46 g/dL (95% CI 12.28–12.65) versus 12.9 g/dL (95% CI 12.52–13.28), *p* < 0.05; Table 2).

**Table 2:** Age distribution and biochemical parameters in 44 children with infantile nephropathic cystinosis and 97 CKD controls

	INC			CKD			
Repeated measurements	Estimated marginal mean (95% CI)	Min.–Max	*N*	Estimated marginal mean (95% CI)	Min.–Max	*N*	*p*-value
Mean age, years	9.94 (9.09–10.78)	2.28–17.90	9	9.13 (8.57–9.70)	2.09–17.93	221	0.121
Cohort 2–6 yrs	4.95 (4.28–5.63)	2.28–6.99	32	4.57 (4.11–5.02)	2.09–6.96	77	0.342
Cohort 7–12 yrs	9.82 (9.02–10.63)	7.33–12.82	29	10.02 (9.60–10.44)	7.00–12.99	100	0.649
Cohort 13–17 yrs	15.21 (14.54–15.88)	13.05–17.90	31	15.15 (14.65–15.65)	13.08–17.93	44	0.881
eGFR, mL/min per 1.73 m^2^	65.84 (59.70–71.98)	6.58–137.67	91	47.89 (44.08–51.70)	9.01–144.87	220	0.000
HCO_3_, mmol/L	22.84 (22.34–23.34)	16.7–29.20	74	22.86 (22.56–23.17)	16.70–30.00	213	0.946
Hemoglobin, g/dL	12.90 (12.52–13.28)	8.10–17.90	89	12.46 (12.28–12.65)	8.00–17.80	219	0.042
Sodium, mmol/L	139.86 (139.21–140.52)	132.00–151.00	90	140.41 (140.10–140.71)	133.00–149.00	219	0.136
Potassium, mmol/L	3.89 (3.78–3.99)	2.37–5.84	91	4.54 (4.47–4.61)	3.27–5.92	219	0.000
Calcium, mmol/L	2.28 (2.25–2.31)	1.84–2.55	79	2.37 (2.36–2.39)	1.90–2.97	208	0.000
Calcium, *z*-score	− 0.88 (− 1.12 to − 0.64)	− 4.74–1.40	79	− 0.08 (− 0.24–0.07)	− 4.95–4.87	208	0.000
Phosphate, mmol/L	1.32 (1.27–1.38)	0.67–2.39	83	1.46 (1.43–1.50)	0.92–2.11	208	0.000
Phosphate, *z*-score	− 1.10 (− 1.47 to − 0.73)	− 4.49–5.16	83	− 0.49 (− 0.66– − 0.33)	− 3.08–3.14	208	0.003
PTH, ng/L	106.61 (77.69–135.54)	4.10–623.40	81	97.35 (87.69–107.02)	5.10–607.92	196	0.548

*N* describes the number of valid measurements for respective parameter out of overall patient measurements, i.e., across all age subgroups. Repeated measurements per patient were evaluated in linear-mixed models

### Age-related changes in anthropometric parameters

**Fig. 1:**
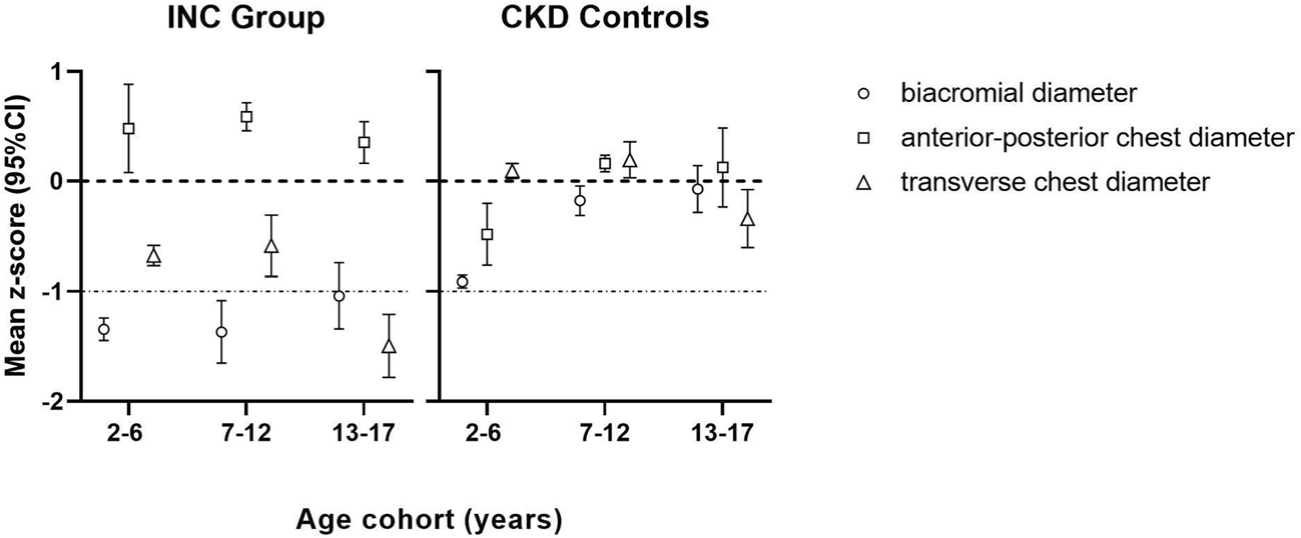
Mean *z*-scores of biacromial diameter (circle), anterior–posterior chest diameter (square), and transverse chest diameter (triangle) of 44 patients with infantile nephropathic cystinosis (INC) and 97 CKD controls. Data are presented for three age cohorts (2–6, 7–12, and 13–17 years) as age- and sex-dependent *z*-scores. Error bars represent 95% confidence intervals. Dotted lines are for illustrative purposes only, representing changes in patterns between the three observed parameters

**Fig. 2:**
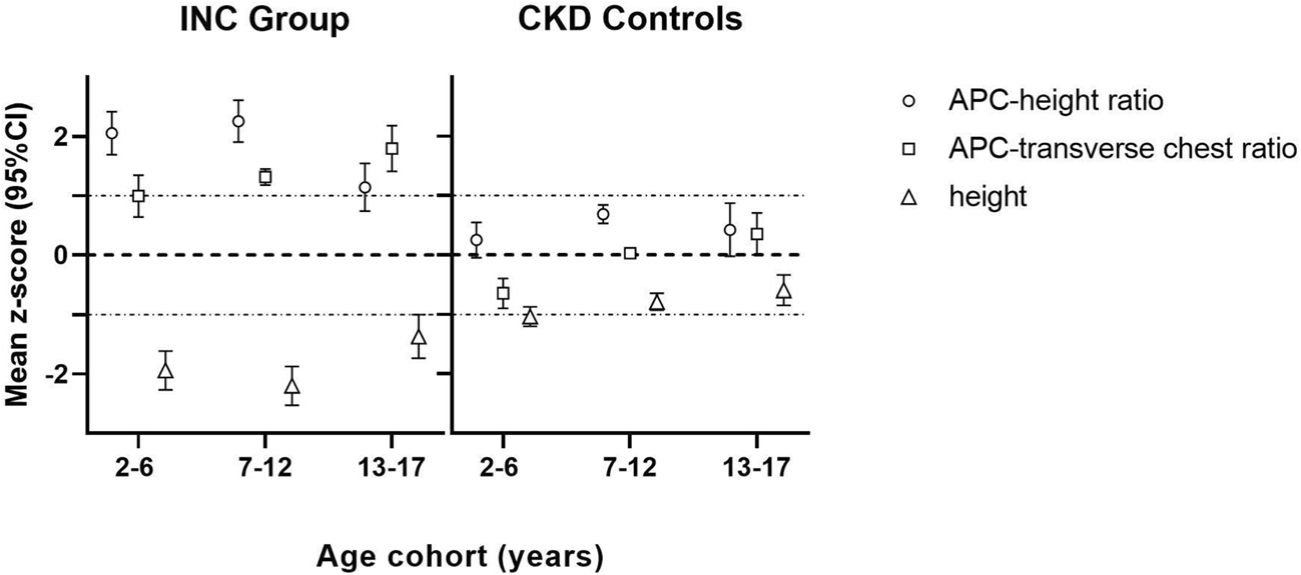
Mean *z*-scores of anterior–posterior chest / height ratio (APC-height ratio; circle) and anterior–posterior chest / transverse chest ratio (APC-transverse chest ratio; square), and body height (triangle) of 44 patients with infantile nephropathic cystinosis (INC) and 97 CKD controls. Data are presented for three age cohorts (2–6, 7–12, and 13–17 years) as age- and sex-dependent *z*-scores. Error bars represent 95% confidence intervals. Dotted lines are for illustrative purposes only, representing changes in patterns between the three observed parameters

Across all age groups, thoracic depth (anterior–posterior chest diameter) and width (transverse chest diameter) significantly differed between patients with INC and CKD controls, as thoracic depth z-scores were consistently increased (each *p* < 0.001) in patients with INC compared to CKD controls, while thoracic width was consistently decreased (each *p* < 0.01), resulting in a pronounced chest disproportion as opposed to the more homogenous *z*-scores observed in CKD controls (Fig. 1). Similarly, shoulder width (biacromial diameter) was likewise lower in patients with INC compared to CKD controls and reached the level of statistical significance at ages 7–12 and 13–17 years (each *p* < 0.001). In general, the degree of chest disproportion was more pronounced in patients with INC compared to CKD controls, irrespective of age. Thoracic disproportion in patients with INC is further evidenced by markedly increased *z*-scores for APC-height and APC-transverse chest diameter ratios (Fig. 2). Both ratio *z*-scores were significantly higher in patients with INC compared to CKD controls, starting in the youngest age group (2–6 years) and across all observed age groups (each *p* < 0.05; Fig. 2).

Standardized APC-height ratio was the parameter that most intensely distinguished patients with INC and CKD in the youngest group (INC 2.06 *z*-score vs. CKD 0.25 *z*-score; *p* < 0.001) and reached its maximum at 7–12 years (INC 2.26 *z*-score vs. CKD 0.69 *z*-score; *p* < 0.001). Afterwards, standardized APC-height ratio in INC patients significantly decreased (*p* < 0.001), as overall height *z*-scores in INC patients significantly increased from the 7–12- to the 13–17-year-old age group (*p* < 0.01). Hence, in the oldest group, APC-transverse chest ratio *z*-scores differed more intensely between groups (INC 1.80 *z*-score vs. CKD 0.36 *z*-score; *p* < 0.001), after exhibiting a continuously significant increase across from each age group to the next and overall, from youngest to oldest group (each *p* < 0.05). Taken together, standardized APC-height ratio appeared to be the most sensitive measure of thoracic disproportion in early childhood, whereas standardized APC-transverse chest ratio steadily increased into late adolescence. Although *z*-scores of both observed measures of thoracic disproportion remained markedly lower in CKD controls compared to INC patients (each *p* < 0.05), both exhibited a similar development across age groups with a maximum APC-height ratio z-score at ages 7–12 years and an increase in APC-transverse chest ratio *z*-scores from youngest to oldest patients (*p* < 0.05) (Fig. 2).

### Biochemical predictors of chest configuration

In patients with INC, standardized APC-height ratio exhibited significant associations with clinical parameters only before adolescence (ages 2–12 years, Table 3). Factors related to tubular dysfunction were associated with APC-height ratio *z*-scores: lower sodium (ages 2–6 years; *p* < 0.05), lower phosphate (ages 2–6 years and 7–12 years) and higher PTH (ages 7–12 years) were significantly associated with more intense increase in APC-height ratio *z*-score (each *p* < 0.05). Lower bicarbonate levels were associated with higher standardized APC-height ratio at ages 7–12 years (*p* < 0.05).

**Table 3:** Linear mixed-effects models of clinical determinants of chest proportions, i.e. anterior–posterior chest diameter/height ratio (APC-height ratio), in three separate age groups of children with infantile nephropathic cystinosis and CKD controls

	INC group			CKD group		
Parameter	2–6 years *N* = 32	7–12 years *N* = 29	13–17 years *N* = 31	2–6 years *N* = 77	7–12 years *N* = 100	13–17 years *N* = 44
Intercept	23.42 (6.61 to 40.24)^b^	8.53 (− 10.36 to 27.41)	0.49 (− 17.38 to 18.35)	− 10.83 (− 34.96 to 13.30)	8.50 (− 4.37 to 21.36)	− 2.29 (− 35.97 to 31.38)
Hemoglobin	0.17 (− 0.30 to 0.63)	0.33 (− 0.08 to 0.74)	− 0.08 (− 0.33 to 0.17)	− 0.38 (− 0.73 to − 0.03)^a^	− 0.03 (− 0.20 to 0.13)	0.16 (− 0.19 to 0.52)
HCO_3_	0.05 (− 0.13 to 0.24)	− 0.29 (− 0.50 to − 0.08)^a^	0.11 (− 0.13 to 0.35)	− 0.15 (− 0.41 to 0.10)	− 0.10 (− 0.19 to 0.00)	− 0.08 (− 0.29 to 0.13)
Sodium	− 0.17 (− 0.30 to − 0.04)^a^	− 0.04 (− 0.17 to 0.09)	− 0.02 (− 0.14 to 0.10)	0.12 (− 0.06 to 0.29)	− 0.04 (− 0.13 to 0.06)	− 0.02 (− 0.25 to 0.21)
Potassium	0.10 (− 0.65 to 0.85)	0.57 (− 0.53 to 1.68)	0.39 (− 0.67 to 1.45)	0.74 (− 0.20 to 1.67)	− 0.07 (− 0.49 to 0.35)	1.06 (− 0.00 to 2.11)
Calcium	− 0.16 (− 0.67 to 0.35)	0.43 (− 0.24 to 1.11)	0.21 (− 0.21 to 0.64)	0.30 (− 0.14 to 0.73)	− 0.12 (− 0.29 to 0.06)	0.22 (− 0.31 to 0.76)
Phosphate	− 0.37 (− 0.73 to − 0.02)^a^	− 0.63 (− 1.06 to − 0.20)^b^	0.21 (− 0.03 to 0.46)	− 0.08 (− 0.59 to 0.44)	− 0.03 (− 0.23 to 0.16)	0.26 (− 0.10 to 0.63)
PTH (*100)	0.49 (− 0.21 to 1.19)	0.47 (0.05 to 0.89)^a^	0.09 (− 0.27 to 0.44)	− 0.13 (− 1.12 to 0.85)	0.15 (− 0.26 to 0.56)	− 0.02 (− 0.62 to 0.57)
eGFR (*100)	− 2.71 (− 4.45 to − 0.97)^b^	− 1.89 (− 4.73 to 0.94)	0.97 (− 1.39 to 3.34)	− 0.45 (− 2.58 to 1.68)	0.20 (− 0.89 to 1.28)	0.73 (− 2.81 to 4.27)

Data are presented as *β*-values (95% confidence intervals)

*N* describes the number of measurements per age subgroup; patients were examined multiple times within age groups; For estimations of effects of calcium and phosphate, z-scores were used

Algebraic sign in *β*-values expresses positive ( +) or negative (–) association e.g. “-0.17” for sodium in the 2–6 year old INC group represents lower sodium levels to be associated with higher APC-height ratio

^a^ p < 0.05; ^b^ p < 0.01

Within early childhood (ages 2–6 years), lower eGFR values were generally significantly associated with a higher APC-height ratio *z*-score (*p* < 0.01, Table 3). Further, it was found that, within the youngest age group (2–6 years), patients exhibited significantly less-progressive thoracic deformation with age when eGFR values were higher than the estimated marginal mean (90.7 mL/min/1.73 m^2^) of the respective group (*β*-value − 0.004 (95% CI –0.006 to − 0.002), *p* < 0.01).

As opposed to the limited early age range during which significant associations were present for standardized APC-height ratio, all INC age groups showed significant associations with assessed clinical parameters for APC-transverse ratio *z*-score (Table 4). In the youngest INC group, attributes of Fanconi syndrome (lower levels of sodium and phosphate) exhibited significant associations with APC-transverse chest ratio *z*-score elevation (each *p* < 0.01).

**Table 4:** Linear mixed-effects models of clinical determinants of chest proportion, i.e., anterior–posterior chest diameter/transverse chest diameter ratio (APC-transverse ratio), in three separate age groups of children with infantile nephropathic cystinosis and CKD controls

	INC group			CKD group		
Parameter	2–6 years *N* = 32	7–12 years *N* = 29	13–17 years *N* = 31	2–6 years *N* = 77	7–12 years *N* = 100	13–17 years *N* = 44
Intercept	15.71 (2.94 to 28.48)	13.90 (− 7.43 to 35.22)	4.13 (− 20.22 to 28.47)	− 1.34 (− 23.84 to 21.16)	− 2.01 (− 17.71 to 13.69)	− 22.19 (− 52.38 to 8.00)
Hemoglobin	0.13 (− 0.22 to 0.48)	0.28 (− 0.18 to 0.74)	− 0.37 (− 0.71 to − 0.03)^a^	− 0.29 (− 0.62 to 0.03)	0.00 (− 0.19 to 0.20)	0.32 (− 0.00 to 0.64)
HCO_3_	− 0.06 (− 0.20 to 0.08)	− 0.32 (− 0.55 to − 0.08)^a^	0.19 (− 0.14 to 0.52)	− 0.07 (− 0.30 to 0.17)	− 0.04 (− 0.15 to 0.08)	− 0.11 (− 0.29 to 0.08)
Sodium	− 0.15 (− 0.25 − 0.05)^b^	− 0.03 (− 0.18 to − 0.12)	0.04 (− 0.12 to 0.21)	0.01 (− 0.15 to 0.18)	0.02 (− 0.01 to 0.14)	0.14 (− 0.06 to 0.34)
Potassium	1.38 (0.81 to 1.94)^b^	− 0.99 (− 2.24 to 0.25)	− 1.39 (− 2.83 to 0.06)	0.68 (− 0.18 to 1.55)	− 0.03 (− 0.54 to 0.49)	0.32 (− 0.63 to 1.27)
Calcium	0.09 (− 0.30 to 0.48)	− 0.29 (− 1.05 to 0.47)	0.66 (0.08 to 1.23)^a^	0.18 (− 0.23 to 0.58)	− 0.24 (− 0.46 to − 0.03)^a^	0.10 (− 0.38 to 0.58)
Phosphate	− 0.53 (− 0.69 to − 0.16)^b^	− 0.37 (− 0.85 to 0.11)	− 0.05 (− 0.38 to 0.29)	0.01 (− 0.47 to 0.49)	0.03 (− 0.21 to 0.26)	0.42 (0.09 to 0.74)^a^
PTH (*100)	0.51 (− 0.02 to 1.04)	0.15 (− 0.32 to 0.62)	− 0.36 (− 0.85 to 0.13)	− 0.11 (− 1.03 to 0.82)	0.07 (− 0.43 to 0.57)	− 0.22 (− 0.75 to 0.31)
eGFR (*100)	0.00 (− 0.13 to 0.13)	− 3.28 (− 6.49 to − 0.08)^a^	− 3.70 (− 6.92 to − 0.47)^a^	1.60 (− 0.38 to 3.59)	0.06 (− 1.26 to 1.38)	0.78 (− 2.39 to 3.96)

Data are presented as *β*-values (95% confidence intervals)

N describes the number of measurements per age subgroup; patients were examined multiple times within age groups; for estimations of effects of calcium and phosphate, z-scores were used. Algebraic sign in *β*-values expresses positive ( +) or negative (–) association, e.g., “ − 0.17” for sodium in the 2–6-year-old INC group represents lower sodium levels to be associated with higher APC-height ratio

^a^*p* < 0.05

^b^*p* < 0.01

In the two older groups, however, lower eGFR (ages 7–12 and 13–17 years; each *p* < 0.05) and lower serum bicarbonate levels (ages 7–12 years; *p* < 0.05) were associated with higher standardized APC-transverse chest ratio.

Additional significant associations within the assessed cluster of variables were visible, with lower hemoglobin values at 7–12 years, higher potassium levels at 2–6 years and higher calcium levels at 13–17 years (each *p* < 0.05) all being associated with standardized APC-transverse chest ratio.

In contrast, only a few significant associations between measures of chest proportions and clinical parameters were noted in CKD controls. APC-height ratio *z*-score was associated with blood hemoglobin (ages 2–6 years), while APC-transverse chest ratio *z*-score was associated with serum calcium (ages 7–12 years) and serum phosphate (ages 13–17 years, each *p* < 0.05; Tables 3 and 4).

## Discussion

This study revealed marked alterations in chest configuration in children with INC characterized by increased chest depth that is distinct from age-matched children with CKD stemming from other causes. This underlines the multisystem implications of the systemic lysosomal storage disease, especially pulmonary insufficiency that was previously reported in 69% of adult patients with INC [29]. Adult restrictive lung disease of extraparenchymal origin with reports of conical chest shape [7, 9] is now shown to be preceded by increased chest depth from childhood onward, which may even contribute to the later clinical presentation, especially as such a chest shape has previously been shown to be associated with poorer prognosis in children facing respiratory stress, i.e., infection [30].

Patients with cystinosis presented with substantially reduced shoulder and chest width, as well as height, but increased chest depth, resulting in a marked increase in APC-height and APC-transverse chest ratio, in *z*-scores (> 1) and in comparison to their peers with CKD. During early childhood, standardized APC-height ratio appeared as the most pronounced measure of chest disproportion in patients with INC, reaching its maximum at 7–12 years, and was less pronounced in adolescent age likely due to the observed parallel increase in height attributed to increased leg growth [10]. Standardized APC-transverse ratio, on the other hand, exhibited a sustained continuous increase, suggesting disproportion within the horizontal plane of the ribcage of INC patients to intensify with increasing age. This stresses the importance of observing different ratios as indicators of thoracic disproportion at different stages of childhood development.

Accordingly, in the multivariate analysis, APC-height ratio *z*-score exhibited significant associations with clinical parameters only at pre-adolescent ages (6–12 years), further underlining the plasticity in that ratio during early childhood. Its elevation was associated with the degree of Fanconi syndrome (e.g., hypophosphatemia, low bicarbonate levels). Both hypophosphatemia and acidosis are main causes of rickets resulting in impaired apoptosis of hypertrophic chondrocytes and consecutive widening of the growth plates in long bones and costal arches, and in severe cases of for example nutritional rickets, resulting in pectus carinatum, and thus increased chest depth [31, 32]. Despite those implications of Fanconi syndrome for ribcage development, our results suggest further factors to be at play. It is generally assumed that Fanconi syndrome is the first clinically apparent sign of INC, preceding the decline of eGFR. However, present results show that, despite a relatively preserved eGFR at a young age (2–6 years) [10], lower eGFR values in early childhood are significantly associated with increased APC-height ratio *z*-scores and thus the degree of chest disproportion [1, 2]. Furthermore, the observed progressive increase with age in APC-height ratio *z*-scores within the youngest age group was significantly less intense when patients exhibited eGFR values above estimated marginal mean. This highlights the importance of early diagnosis and commencement of cysteamine therapy in these patients, which has been shown to allow for the physiologically expected increase in eGFR during infancy and to ameliorate progressive CKD at a later age [33]. Whether a direct causal link of reduced glomerular function and Fanconi syndrome with the observed thoracic deformation is present or whether they are merely indicators of disease progression or intensity remains unclear. In addition to the influences of Fanconi syndrome and CKD–MBD (mineral and bone disorder), cystinosis metabolic bone disease (CMBD) is multifactorial and not yet fully understood. Influences of this specific disease entity on the bones, e.g. the bones of the ribcage, include effects of the *CTNS* mutation on the functionality of osteoblasts and osteoclasts, as well as cysteamine toxicity [6, 34–36].

Other factors than the evaluated parameters could contribute to this either directly, e.g., vacuolar myopathy, which has been reported to be associated with intensity of pulmonary dysfunction in INC [7], or on a grander developmental scale. A rather rounded thoracic shape is typically seen in the early infantile period of healthy children [37], changing to a more ovoid shape during the first two years of life, hence leading to a decreasing APC-transverse chest ratio with age [37]. In INC patients, however, elevated thoracic ratio z-scores continue to persist into childhood, suggesting an aberrant thoracic development in the preceding infantile period. Further, hypophosphatemic rickets is speculated to delay ambulation [38–41], which is generally assumed to normally contribute to changes in thoracic geometry and rib orientation through postural changes [37, 42]. Alterations in the growth hormone (GH)–insulin-like growth factor 1 (IGF1) axis and IGF1/GH downstream signaling through malnutrition, insulin, and thyroid hormone deficiency in INC [1, 43–45] might further hinder skeletal muscle growth [46] and bone development [47], as well as the transition between infantile and childhood developmental phases, which is the main timeframe for changes in ribcage geometry [37, 48, 49].

APC-transverse chest ratio *z*-scores were significantly associated with several clinical parameters across all age groups, which is in accordance with its continuous increase across all observed ages. In the youngest group (2–6 years), those were predominantly related to Fanconi syndrome (low serum sodium and phosphate concentrations). Then, a gradual shift occurred, toward complications of INC usually occurring at higher ages, with APC-transverse chest ratio z-score being associated with lower eGFR values from 7–12 years onward and lower hemoglobin values at ages 13–17 years. This age-related shift in determinants seems to develop in parallel to INC disease progression, as predominant tubular dysfunction is the initial hallmark of INC, and loss of glomerular function progresses over time [1, 43]. Surprisingly, a significant negative association between standardized APC-transverse chest ratio and serum potassium levels was seen in the youngest INC group, which may be due to more intense potassium substitution in severe cases of Fanconi syndrome. Further, higher calcium levels were unexpectedly associated with higher APC-transverse chest ratio z-scores in the oldest INC patients. As albumin-corrected calcium was used for calculations and hypocalcemia and proteinuria [39] might introduce discordance between calcium and albumin levels [50, 51], interpretation of this particular association is highly complex and exceeds the scope of this analysis.

In contrast to patients with INC, chest configuration in CKD controls was only mildly affected (values within ± 1.0 *z*-score). The low biacromial diameter that was visible across all ages in CKD controls and even more pronounced in patients with INC is known to be linked to low physical activity and poorer living conditions [12, 13] and is thus likely due to the influences of chronic disease. In the youngest age group in particular, patients with CKD showed a strikingly different chest shape pattern compared to patients with INC, where chest depth was not significantly increased, but reduced, and chest width was the best-preserved parameter. At ages 13–17 years, however, APC-transverse chest ratio *z*-score significantly increased in CKD controls, culminating in a pattern where chest depth was the highest individual parameter, similar to the pattern seen in patients with INC, if far less intense and later in life, possibly as a result of longstanding complications (Fig. 1). Low calcium and higher phosphate were associated with higher APC-transverse chest ratio z-scores at ages 7–12 and 13–17 years, respectively, possibly hinting at rachitic thoracic deformation [14] due to CKD–MBD caused by progressive CKD [52].

Our findings pose questions regarding possible clinical implications of the observed chest shape [7, 9, 29] and highlight the importance of the improvement of childhood development through optimal and early causal treatment with cysteamine. Thus, further research would be beneficial, regarding possible myopathy of the chest wall, assessments of lung function and analyses of the possible impact of cysteamine therapy, e.g., time-averaged weight-related cysteamine dosages or leukocyte cystine levels, on chest configuration. Further, non-invasive positive pressure ventilation has been reported to alleviate symptoms of restrictive respiratory dysfunction in adult patients with INC [8, 9]. As an increase in chest depth has previously been described to be linked to worse outcomes in respiratory infection [30], and as such has been found to be the leading cause of respiratory mortality in patients with INC [29], earlier consideration of this treatment option, as well as treatment for Fanconi syndrome, may be beneficial for affected patients and needs to be further evaluated. An early evaluation of patients with INC regarding respiratory function may also be useful. Those factors, however, exceed the scope of this present analysis, which provided the initial description of an INC-specific thoracic disproportion with increased chest depth, which persists into adulthood, and is associated with the degree of tubular dysfunction and CKD.

### Supplementary Information

Below is the link to the electronic supplementary material.

Graphical abstract (PPTX 212 KB)

## Data Availability

The data that support the findings of this study are available on request from the corresponding author. The data are not publicly available due to privacy or ethical restrictions.
