# Supplementary material for: Chest configuration in children and adolescents with infantile nephropathic cystinosis compared with other chronic kidney disease entities and its clinical determinants
Source: Pediatr Nephrol. 2023 Jul 7;38(12):3989–99. doi: 10.1007/s00467-023-06058-x (PMC10584709; doi:10.1007/s00467-023-06058-x)
Supplement: Supplementary file 1 — Graphical abstract (PPTX 212 KB) [file 467_2023_6058_MOESM1_ESM.pptx]

## Slide 1
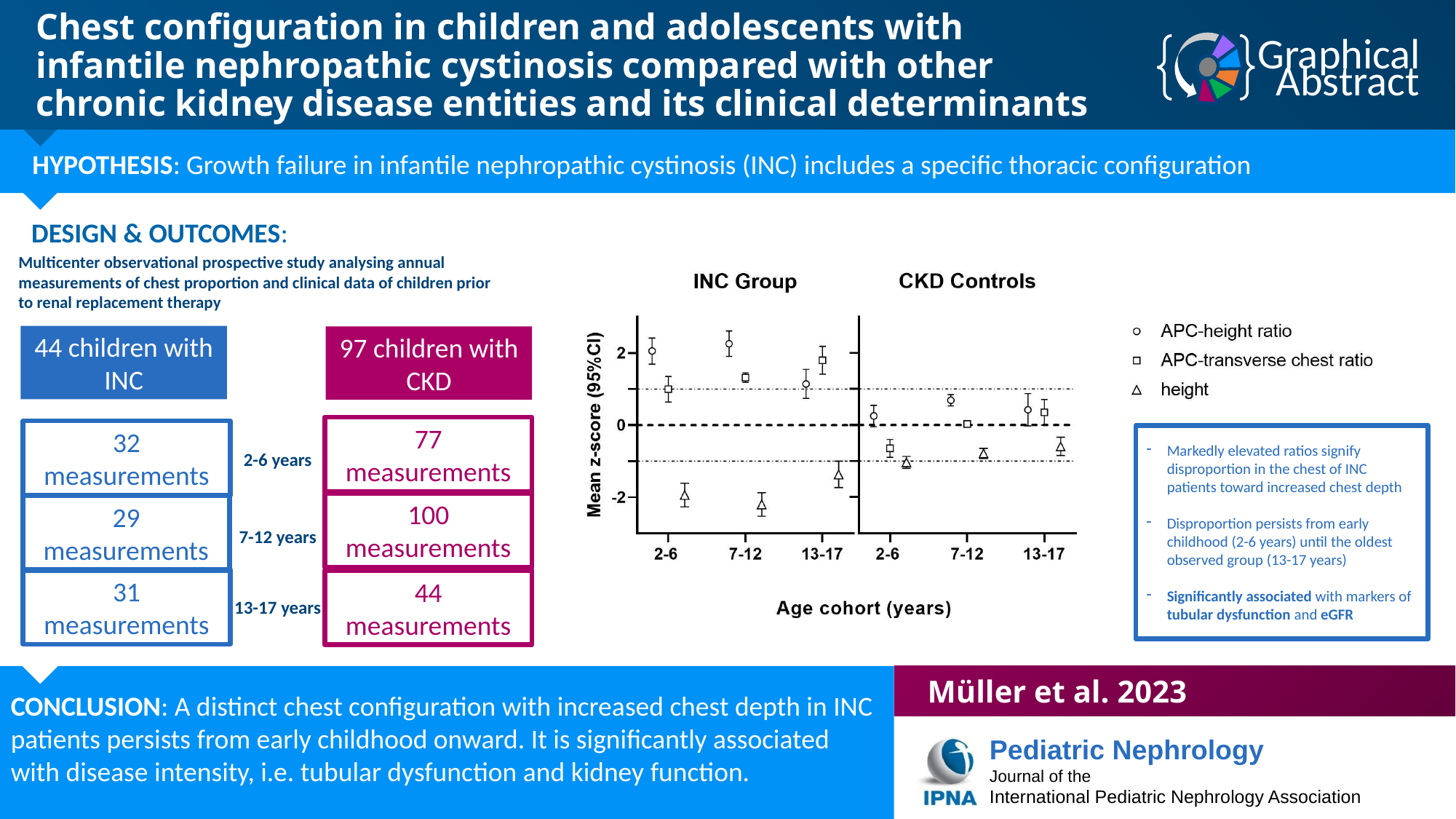

Chest configuration in children and adolescents with infantile nephropathic cystinosis compared with other chronic kidney disease entities and its clinical determinants
HYPOTHESIS: Growth failure in infantile nephropathic cystinosis (INC) includes a specific thoracic configuration
DESIGN & OUTCOMES:
Multicenter observational prospective study analysing annual measurements of chest proportion and clinical data of children prior to renal replacement therapy
44 children with INC
97 children with CKD
77 measurements
32 measurements
Markedly elevated ratios signify disproportion in the chest of INC patients toward increased chest depth
Disproportion persists from early childhood (2-6 years) until the oldest observed group (13-17 years)
Significantly associated with markers of tubular dysfunction and eGFR
2-6 years
100 measurements
29 measurements
7-12 years
31 measurements
44 measurements
13-17 years
Müller et al. 2023
CONCLUSION: A distinct chest configuration with increased chest depth in INC patients persists from early childhood onward. It is significantly associated with disease intensity, i.e. tubular dysfunction and kidney function.
